# C-856-01. Bridging Gaps in Burn Care: Understanding Outcomes in Survivors of Domestic Violence - Scoping Review

**DOI:** 10.1093/jbcr/irag033.093

**Published:** 2026-04-06

**Authors:** Sukhmeet S Sachal, Abhishek Achunair, Syeda Taliya Rizvi, Noor Qureshi, Alla Iansavitchene, Lloy Wylie, Katie Garland, Tanya DeLyzer, Kimberley T Jackson, Rae Spiwak, Aishwarya Raj, Caitlin Symonette, Sarvesh Logsetty

**Affiliations:** Western University, London, Ontario; Western University, London, Ontario; Schulich School of Medicine and Dentistry - Western University, London, Ontario; Schulich School of Medicine and Dentistry - Western University, London, Ontario; London Health Sciences Centre Research Institute, London, Ontario; Western University, London, Ontario; Western University, London, Ontario; Western University, London, Ontario; Western University, London, Ontario; University of Manitoba, Winnipeg, MB; All India Institute of Medical Sciences, New Delhi, India; Children's Hospital, London Health Sciences Center, London, Ontario; Manitoba Firefighters Burn Unit, Winnipeg, MB

## Abstract

**Introduction:**

Burn injuries are a leading cause of global morbidity and mortality, with survivors of domestic violence (DV) facing uniquely severe and complex outcomes. Despite their prevalence, DV-related burns remain underrecognized in clinical research. This scoping review synthesizes evidence on the prevalence, injury patterns, and clinical, surgical, and psychosocial consequences of DV-related burns to highlight gaps essential for future intervention.

**Methods:**

Following PRISMA-ScR guidelines, MEDLINE, Embase, APA PsycInfo, and CINAHL were queried for studies published between January 1, 2000, and January 1, 2025. Eligible studies included observational designs, case series, and case reports analyzing outcomes of DV-related burns. Findings were extracted and summarized descriptively.

**Results:**

From 1567 screened records, 56 met inclusion. Survivors of DV-related burns consistently presented with more extensive injuries, with 10 studies reporting higher Total Body Surface Area (TBSA) burns (median 13–27%). Twelve studies described distinct injury distributions, with up to 78% involving head, neck, and facial involvement. Seven studies noted additional trauma such as fractures, blunt injury, or strangulation. Treatment delays were observed in eight studies; one documented delays in 38% of DV cases versus 6% of controls. Survivors required substantially greater healthcare resources: nine studies showed hospitalizations prolonged 1.5–3 times, while six documented longer ICU stays (median 7.8 vs. 4.4 days) and increased ventilator dependence. Mortality disparities were substantial: kerosene-flame assaults reached mortality rates up to 97%, and hazard ratios of death were 4.67 times greater than controls. Seventeen studies emphasized the overwhelming surgical burden, with survivors often undergoing 10 or more excisions, grafts, and staged reconstructions. Long-term outcomes included elevated rates of hypertrophic scarring, contractures, PTSD, depression, and social ostracism.

**Conclusions:**

DV-related burns are consistently more severe, delayed in presentation, and associated with higher surgical, clinical, and psychosocial burden compared to non-DV burns. These findings illuminate a critical gap in burn care and call for trauma-informed, multidisciplinary models to improve outcomes for this vulnerable population. Recognizing these injury patterns can transform clinical pathways, improve survival, and support holistic recovery.

**Applicability of Research to Practice:**

For clinicians, these data illuminate predictors that can trigger earlier screening for DV, facilitate timely interventions, and guide surgical and psychosocial care planning. For policymakers and burn units, the evidence supports urgent investment in coordinated models that bridge clinical, social, and community-based care. By translating these insights into practice, we can close an unacceptable gap in burn care equity.

**Funding for the study:**

Foundation Funding $5000.

